# Microendoscopy detects altered muscular contractile dynamics in a mouse model of amyotrophic lateral sclerosis

**DOI:** 10.1038/s41598-019-56555-z

**Published:** 2020-01-16

**Authors:** Xuefeng Chen, Gabriel N. Sanchez, Mark J. Schnitzer, Scott L. Delp

**Affiliations:** 1 Department of Mechanical Engineering, Stanford University, Stanford, CA 94305 USA; 2 Department of Biology, Stanford University, Stanford, CA 94305 USA; 3 Department of Applied Physics, Stanford University, Stanford, CA 94305 USA; 4 Howard Hughes Medical Institute, Stanford University, Stanford, CA 94305 USA; 5 Department of Bioengineering, Stanford University, Stanford, CA 94305 USA; 6 Enspectra Health, Mountain View, CA 94040 USA

**Keywords:** Electrophysiology, Microendoscopy, Amyotrophic lateral sclerosis

## Abstract

Amyotrophic lateral sclerosis (ALS) is a fatal disease involving motor neuron degeneration. Effective diagnosis of ALS and quantitative monitoring of its progression are crucial to the success of clinical trials. Second harmonic generation (SHG) microendoscopy is an emerging technology for imaging single motor unit contractions. To assess the potential value of microendoscopy for diagnosing and tracking ALS, we monitored motor unit dynamics in a B6.SOD1G93A mouse model of ALS for several weeks. Prior to overt symptoms, muscle twitch rise and relaxation time constants both increased, consistent with a loss of fast-fatigable motor units. These effects became more pronounced with disease progression, consistent with the death of fast fatigue-resistant motor units and superior survival of slow motor units. From these measurements we constructed a physiological metric that reflects the changing distributions of measured motor unit time constants and effectively diagnoses mice before symptomatic onset and tracks disease state. These results indicate that SHG microendoscopy provides a means for developing a quantitative, physiologic characterization of ALS progression.

## Introduction

ALS is a paralytic, fatal neurodegenerative disease characterized by a progressive loss of motor neurons^1^. The first approved drug to treat ALS, riluzole, which only prolongs survival by a few months^2^, was approved over 20 years ago. Since then, over 50 clinical trials^3^ have resulted in only 1 new recently approved drug^4^, edaravone, whose effect on survival has yet to be studied. Development of effective treatments has suffered from a lack of methods for early diagnosis and sensitive monitoring of the disease^5^. Patients enter clinical trials at late disease stages that may be too advanced to gain therapeutic benefit, and subtle improvements might be undetectable. Sensitive diagnostics are needed to reduce diagnostic delay so that earlier-stage patients can be identified and participate in clinical trials.

To assess if a therapy is effective, it is also important to have biomarkers of disease progression. Currently, the Revised ALS Functional Rating Score (ALSFRS-R) — a numerical score from 0–48 based on assessments of bulbar, limb, and respiratory function^6^ — is the predominant clinical method for measuring disease progression. This score is subjective and a sum of multiple metrics, some of which are highly correlated, making changes in the ALSFRS-R score hard to relate to changes in disease state^7^. As new candidate treatments enter clinical trials, the field needs quantitative diagnostics that reflect the neurophysiologic changes in ALS, provide accurate assessments of disease progression, and inform treatment development and usage.

To meet this challenge, we examined whether second harmonic generation (SHG) microendoscopy might detect the physiological changes that occur in ALS. SHG microendoscopy is a minimally invasive approach to imaging the twitch contractions of individual motor units in live patients^8^. Myosin filaments within muscle sarcomeres are capable of second harmonic generation and move in response to motor neuron activation, thus providing a means to measure twitch dynamics in live humans and animals. Previous histological studies of ALS mouse models showed that fast-fatigable (FF) motor neurons die off before overt symptoms appear; by comparison, fast-fatigue-resistant (FR) motor neurons die at symptom onset, whereas slow (S) motor neurons survive the longest and are the most likely to reinnervate other denervated fibers^9–11^. With the aim of developing a method to diagnose and track progression of ALS, we hypothesized that this heterogeneous progression of motor neuron denervation induces a change in motor unit composition towards motor units with slower contractile dynamics that might be empirically detectable by SHG microendoscopy. In healthy individuals, muscles with different fiber type compositions resulted in differently measured contractile times, so we reasoned that clinical differences should also be detectable. If this hypothesis was correct and the technique detected differences between healthy and a disease model of mice, it could lead to novel clinical means of diagnosing ALS and tracking the disease in patients based on their abnormal neuromuscular dynamics.

Here, we demonstrate that SHG microendoscopy is able to detect changes with progression of ALS disease. To do so, we used SHG microendoscopy to characterize motor unit twitch contractile dynamics in the B6.SOD1G93A mouse model of ALS^12^. We imaged single motor unit muscle twitches in these mice, from the pre-symptomatic to the disease end stages, and we compared the measured contractile time constants to those determined in age-matched wild-type (WT) mice. Starting in pre-symptomatic disease stages, there were notable differences between the two groups, and greater differences arose later in disease progression. Hence, microendoscopy measurements of motor unit contractile dynamics are a means of studying, diagnosing and tracking the disease in a mouse model and pave the way for human studies.

## Results

### Visualizing the contractile dynamics of single motor units in live mice

**Figure 1:**
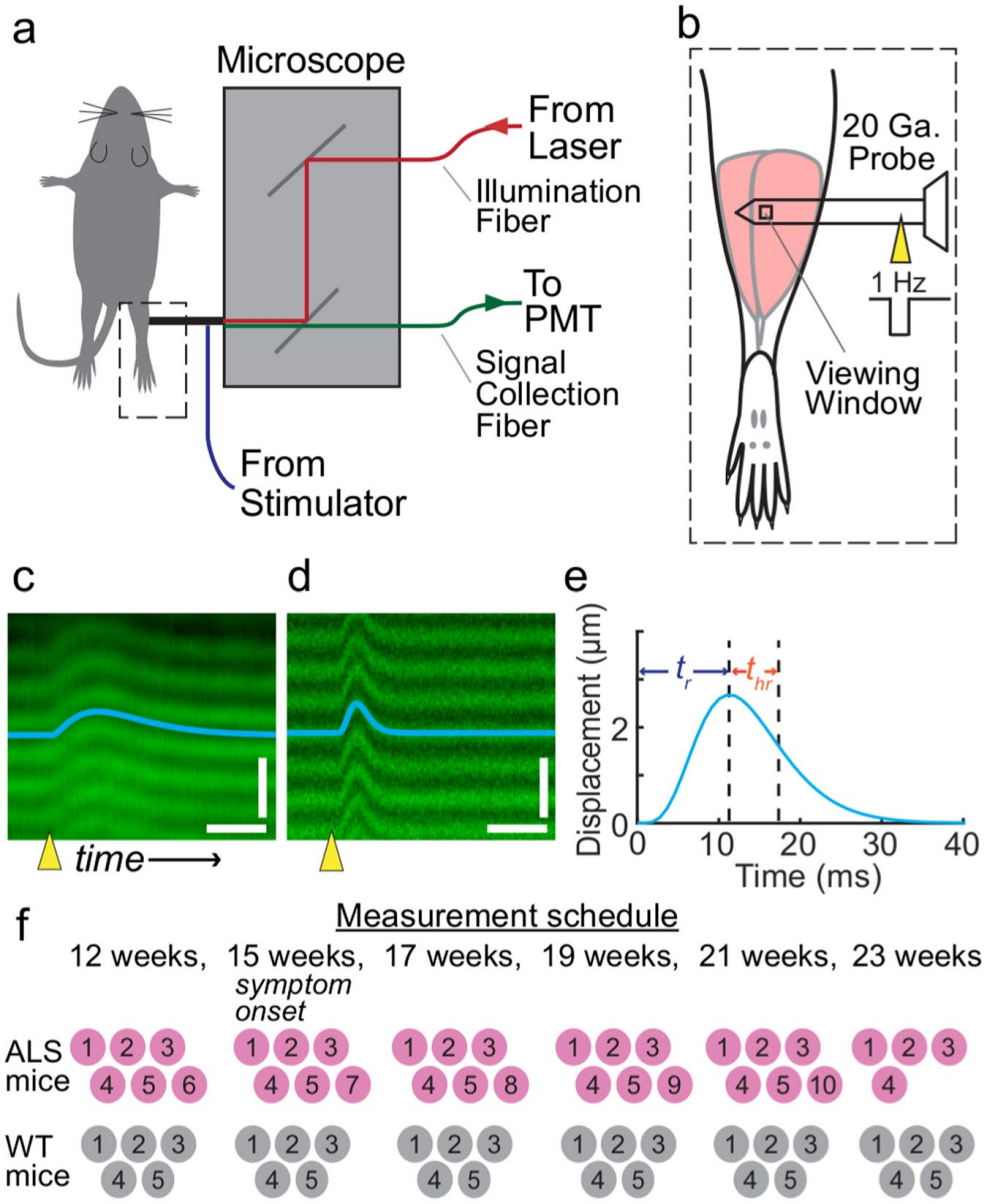
Visualization of single motor twitches by using SHG imaging. (**a**) A Yb-ion laser delivers ultrashort-pulsed, near-infrared illumination to the handheld SHG microscope via an optical illumination fiber. The microscope directs the laser light into a 20-gauge microendoscope probe. The probe has a microprism near its tip for imaging in the direction perpendicular to the probe’s axis and is inserted into the muscle of interest. SHG signals return through a signal collection optical fiber, which routes the signals to a photomultiplier tube (PMT) for detection. An electrical stimulator is connected to the probe and delivers current that is used to stimulate the muscle. (**b**) We performed SHG microendoscopy in the gastrocnemius muscle. (**c,d**) To visualize motor unit twitches, we swept the laser illumination in a line-scanning pattern of 1.09 kHz across the muscle tissue, yielding space-time plots revealing the twitch time courses of the muscle’s individual sarcomeres. The examples shown are averaged images from B6.SOD1G93A, (**c**), and wild-type mice, (**d**). Yellow triangle marks onset of the electrical stimulation. Blue line is the computationally extracted twitch time course. Scale bars: 30 ms horizontal, 5 µm vertical. (**e**) Example twitch time course fit from (**d**). The twitch rise time, *t*_*r*,_ is the duration from stimulation delivery (0 ms) to peak sarcomere displacement. The half-relaxation time, *t*_*hr*_, is the time from peak displacement to half of peak displacement. (**f**) We imaged B6.SOD1G93A and wild-type mice at 6 time points. The numbers denote individual mice and show when each mouse underwent SHG imaging.

Using a mobile SHG microendoscopy system^8^, we visualized the twitch dynamics of individual motor units in the gastrocnemius of B6.SOD1G93A and wild-type mice (Fig. 1a–d). This allowed us to track how the distributions of single motor unit rise and half-relaxation times evolved over weeks in individual mice (Fig. 1e,f). Our measurements began when the mice were 12 weeks old. At this age, the mice were adults but the B6.SOD1G93A mice were still pre-symptomatic. We took measurements every two weeks, until the B6.SOD1G93A mice reached disease end stage (Fig. 1f).

We first verified that the repeated reinsertion of the 20-gauge microendoscope probe into the muscles of the triceps surae of the mouse did not affect the measurements. Across the six measurement time points spaced over 11 weeks, the rise times and half-relaxation times of motor units in wild-type mice remained statistically invariant (p = 0.1 rise times, p = 0.8 half-relaxation times; Gore test for multiple observations; n = 29–40 motor units at each time point, 5 mice). Further, by measuring muscle twitch forces via a tendon transducer, we found that these forces were unaltered by insertion and removal of the probe, consistent with the probe causing minimal tissue trauma (Supplementary Fig. 1).

### Distributions of motor unit time constants in wild-type and B6.SOD1G93A mice

**Figure 2:**
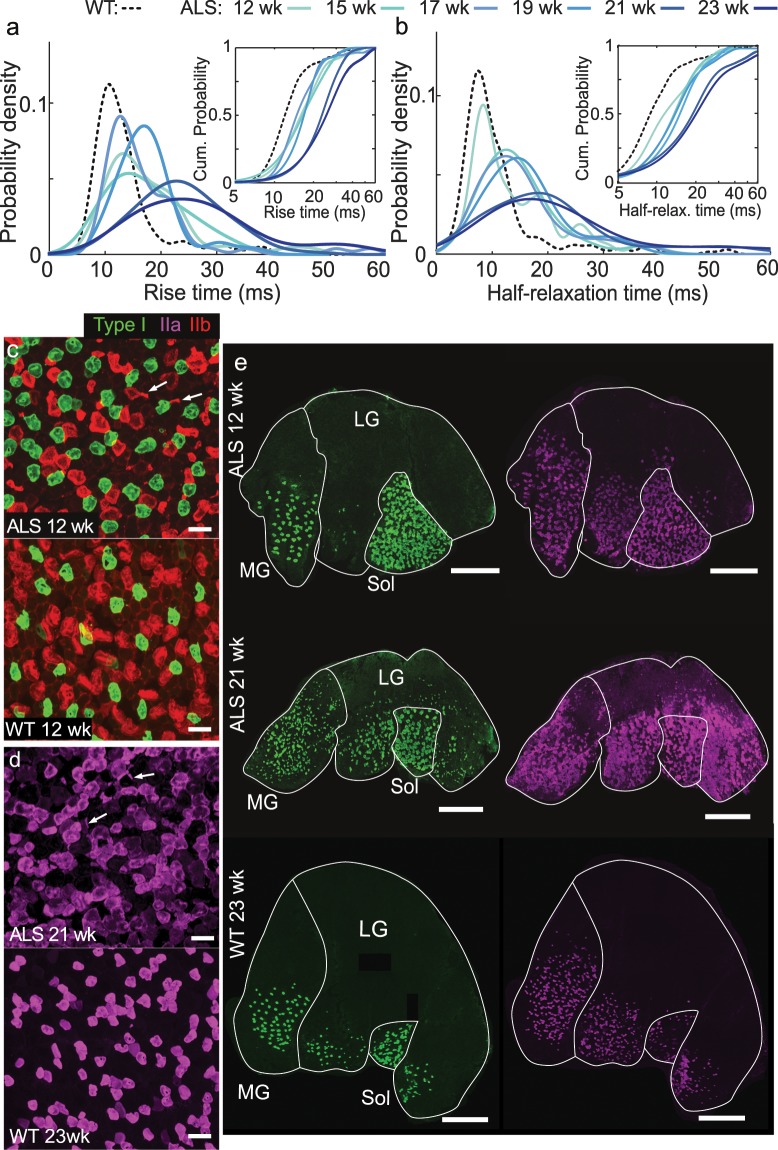
Twitch rise times and half-relaxation times increase with ALS progression. (**a,b**) Estimated probability density and cumulative distribution functions via kernel density estimation (insets) of motor unit (**a**) rise times and (**b**) half-relaxation times. We estimated the curves for wild-type (WT) mice based on all twitches acquired from the WT mice across from all six time points. We estimated the curve for the B6.SOD1G93A mice based on all twitches taken at each individual time point. In the B6.SOD1G93A mice, the distributions shifted towards longer rise times as the mice aged. Median rise times at 21 and 23 weeks were significantly greater than at 12, 15, 17, and 19 weeks; Half-relaxation times at 21 and 23 weeks were greater than at 12 weeks, and half-relaxation times at 23 weeks were greater than at from 15 weeks (p < 0.05, Dunn’s test with Bonferroni correction for multiple comparisons, n = 30–54 motor units at each time point). The distributions of time constants were slower at each time point for the B6.SOD1G93A mice than for WT mice (p = 4·10^−4^; 7·10^−5^; 7·10^−4^; 4·10^–8^; 2·10^−18^; 2·10^−11^ for rise times, p = 0.01; 6·10^−6^; 3·10^−7^; 6·10^−7^; 6·10^−13^; 4·10^−9^ for half-relaxation times, two sample Kolomogov–Smirnov test, n = 222, 43, 45, 54, 39, 55, 30 motor units in the WT and each time point of B6.SOD1G93A groups). (**c**) Representative histological image from the medial gastrocnemius of a 12-week B6.SOD1G93A mouse and 12-week wild-type mouse. Arrows point to atrophy of type IIb muscle fibers stained with BF-F3 primary antibody from the B6.SOD1G93A mouse. Scale bars 100 µm. (**d**) Representative histological image from the medial gastrocnemius of a 21-week old B6.SOD1G93A mouse and a 23-week wild-type mouse. Arrows point to atrophy of type IIa muscle fibers (SC-71 primary antibody), and large clusters of type IIa fibers indicate fiber type grouping present in the B6.SOD1G93A mouse. Scale bar 100 µm. (**e**) Staining for type I (BA–D5 primary antibody) and type IIa fibers in the medial gastrocnemius (MG), lateral gastrocnemius (LG) and soleus (SOL), of B6.SOD1G93A mice at 12 and 21 weeks shows the proliferation of type I and type IIa fibers into new regions of the muscle, especially the LG. Image from 23 week wild-type mouse shown as comparison for age effects. Scale bar 1 mm.

To compare the dynamics of single motor units in B6.SOD1G93A versus wild-type mice, we examined the statistical distributions of the contractile rise and the half-relaxation times in the gastrocnemius. At all time points, the distributions of rise and half-relaxation time constants from the B6.SOD1G93A mice differed significantly from those of wild-type mice (Fig. 2a,b). Specifically, the B6.SOD1G93A mice had a greater proportion of slow rise and half-relaxation times than the wild-type mice at each time point, indicating a greater proportion of slow motor units. We pooled together statistically invariant data to better estimate these statistical distributions. Within each experimental group (B6.SOD1G93A or wild-type), there were no statistically significant differences among the repeated mice in either the rise or half-relaxation times (p > 0.05; Gore test for multiple observations; n = 3–19 motor units per mouse per time point) so we pooled data from each time point together. Furthermore, contractile time constants from wild-type mice were statistically invariant over time (p > 0.1; Gore test for multiple observations; n = 29–40 total motor units at each time point from 5 mice) so we pooled the data from wild-type mice at different time points.

In the B6.SOD1G93A mice over the 11 weeks of study, median rise and the half-relaxation times of single motor unit twitches become progressively slower (p = 7·10^−7^ for rise times, p = 0.008 for half-relaxation times; Gore test for multiple observations; n = 28–36 total motor units at each time point from 5 mice). In the B6.SOD1G93A mice, the rise times at weeks 12–19 differed from those at weeks 21 and 23, and the half-relaxation times at weeks 12 and 15 differed from those 21 and 23 weeks (Supplementary Fig. 2).

### Histologic evidence of motor neuron loss

To corroborate previous findings of denervation within SOD1G93A mice^9,10^ we performed immunohistochemistry staining for different myosin isoforms to assess the muscle fiber types within the triceps surae muscle group comprising the gastrocnemius and soleus. A B6.SOD1G93A mouse was sacrificed at each time point. At 12 weeks, some type IIb fibers, which are innervated by FF motor neurons, appeared atrophied (Fig. 2c). At 21 weeks, immunostained type IIa and type I fibers were more apparent in tissue sections of the lateral gastrocnemius (Fig. 2d). We observed atrophy of type IIa fibers and type grouping, seen as clustering of round fibers of the same type as opposed to a mosaic pattern. Type grouping is indicative of reinnervation as surviving motor neurons reinnervate recently denervated fibers nearby^13^. Also at 21 weeks, type IIa and type I fibers were more apparent than at 12 weeks in the gastrocnemius (Fig. 2e). These findings support the existence of denervation and reinnveration processes that affect muscle fiber type composition.

### Composite twitch time as a candidate biomarker for diagnosing and monitoring

**Figure 3:**
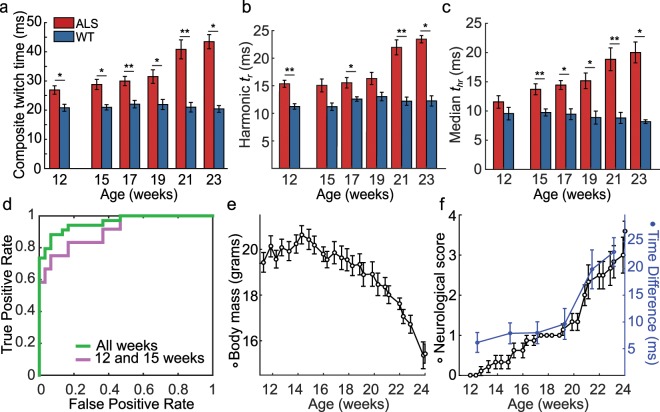
Composite twitch time provides an effective diagnostic and monitoring metric. (**a–c**) Mean ± s.e.m.values of the composite twitch time, **a**, harmonic mean rise time, **b**, and median half-relaxation times, **c**, from individual B6.SOD1G93A and WT mice. *p < 0.05, **p < 0.01, rank sum test. n = 5 WT mice and n = 6 B6.SOD1G93A mice (5 repeated, except at 23 weeks which had 4 B6.SOD1G93A mice. n = 3–19 motor units per mouse; median of 7 per mouse). (**d**) Receiver operating curve attained using the composite twitch time as a metric for distinguishing normalcy and the ALS disease state by using measurements from the B6.SOD1G93A mice taken at the first two time points [Area Under the Cuve (AUC) = 0.91; purple] or all time points (green; AUC = 0.96). (**e,f**) Mean body mass of B6.SOD1G93A mice (**e**), and mean neurological scores, where 1 is initial tremors in B6.SOD1G93A mice (Methods), (**f**), of ALS mice (black filled dots) compared to the difference in composite twitch time between B6.SOD1G93A and all wild-type mice (blue dots). All points are mean ± s.e.m.

Based on the changes in the distributions of motor unit dynamics, we sought a biomarker that would discriminate wild-type mice from B6.SOD1G93A mice prior to overt symptomatic onset and allow quantitative tracking of disease progression. We explored different candidate metrics that could reflect the distribution of the measured twitch contraction waveforms from individual mice, such as the mean, the quartiles, the variance, and skewness (Supplemental Table). From analysis of how these metrics performed in discriminating between the wild-type and B6.SOD1G93A mice, we found the harmonic mean of the rise times and the median of the half-relaxation times provided the greatest discriminatory power. We then constructed a succinct metric that we termed the ‘composite twitch time’ (Fig. 3a), defined for each mouse as the sum of its harmonic-mean rise time (Fig. 3b) and median half-relaxation time (Fig. 3c), as measured across all motor units at a given time point. Defined in this way, the composite twitch time differed statistically between B6.SOD1G93A and wild-type mice at every time point (Fig. 3a).

At 12 weeks, the composite twitch time was 21 ± 1 ms (mean ± s.e.m.) in wild-type mice and 27 ± 1 ms in B6.SOD1G93A mice. The composite twitch time increased with age in B6.SOD1G93A mice (Pearson’s *r* = 0.7). At 21 weeks, the composite twitch time in B6.SOD1G93A mice (41 ± 3 ms) was almost twice that of wild-type mice (21 ± 2 ms). By fitting time-dependent, exponential functions to the harmonic-mean rise times and median half-relaxation times determined in B6.SOD1G93A mice, we found that the two terms in the composite twitch time evolved similarly over the course of disease progression (p = 0.09 and p = 0.25; F-test for half-relaxation time data fitting the model of rise time and for rise time data fitting the model of half-relaxation time, respectively).

We created receiver operating characteristic (ROC) curves to evaluate the discrimination performance of the composite twitch time for distinguishing B6.SOD1G93A from wild-type mice (Fig. 3d). The ROC curve plots the tradeoff between true positive and false positive diagnoses of ALS across a range of threshold values. The area under the ROC curve is the probability that a randomly chosen B6.SOD1G93A mouse will have a greater composite twitch time than a randomly chosen wild-type mouse^14^. Using the data from all six time points, the area under the ROC curve was 0.96 (0.89–0.99, 95% confidence bounds). To evaluate the use of the composite twitch time as an early diagnostic, we constructed another ROC curve using all the data from wild-type mice but only the data from age 12 and 15 weeks in the B6.SOD1G93A mice. This resulted in an area under the ROC curve of 0.91 (0.76–0.98, 95% confidence bounds), indicating the composite twitch is a robust diagnostic even at early time points in the disease.

To compare the time dependence of the composite twitch time with that of overt disease symptoms, we measured the body masses of the mice and assigned behavioral neurological scores (Methods) (Fig. 3e,f). Overt signs of ALS became apparent at 15 weeks in the B6.SOD1G93A mice, as the maximum average body mass occurred near 14.5 weeks of age and half of the B6.SOD1G93A mice started showing tremors when held by the tail at 15 weeks. By comparison, though there were no overt symptoms at 12 weeks, there was already a significant difference in the composite twitch times between B6.SOD1G93A and wild-type mice (Fig. 3a). By 21 weeks, B6.SOD1G93A mice showed weakness (Fig. 3e), including dragging of the toe or no longer extending the foot when lifted by the tail. This accompanied a significant rise in the composite twitch time (p = 0.01 between 12 weeks and 21 weeks, Dunn’s test with Bonferonni correction for multiple comparisons; n = 6 mice). Hence, the composite twitch time revealed early differences between the two groups of mice and increased with progressing symptoms.

## Discussion

Our microendoscopic studies of motor unit twitch contractions revealed progressive changes in contractile dynamics in B6.SOD1G93A mice that likely reflect motor neuron death.

### Measurements of rise times likely reflect the altered distribution of motor unit types

The distributions of rise times that we measured likely represent the motor unit compositions of the muscles studied. The gastrocnemius normally comprises less than 10% S motor units, and the rest are FF and FR motor units^15^. Prior acute studies measured the twitch rise times of individual motor units using force transducers attached to the tendon of the mouse extensor digitorum longus (EDL)^16^, which is made up primarily of FF and FR motor units, and soleus^17^, which is comprised mostly of S motor units. The distributions of rise times that we determined in wild-type mice agree with these historically measured ranges. SHG microendoscopy thus provides a new way to measure motor unit contractile dynamics and motor unit type compositions in specific muscles in non-terminal experiments.

Our histology also presented evidence of muscle fiber denervation and reinnervation leading to altered distributions of motor unit types with progression of disease. At 12 weeks, atrophy of type IIb muscle fibers and slower rise times were noted as FF motor neurons die off. At 21 weeks, some FR motor units degenerated as seen by atrophy of type IIa fibers, while type IIb and type I fibers were more apparent, which we interpreted as re-innervation of fibers by FR and S motor neurons respectively up to this point. Ultimately, the fiber type composition, with different fiber types having different contractile dynamics, affects the timing of measured individual motor units.

A previous study of the medial gastrocnemius of SOD1G93A rats, found there to be a loss of FF motor units and increase in proportion of S motors units in late stage disease, which match our twitch timing observations slowing down over time^18^. Furthermore, the study found motor unit rise times of fast motor unit types increased in early symptomatic animals. This suggests that our measured slower of contraction times in early disease are likely due to a greater percentage of slow motor units and not a slowing of individual motor units. Future studies that better identify the type of motor unit that is under observation through quantitative neuron histology are needed to verify this. Despite these limitations, this work represents a proof of concept that this technology detects a quantitative difference in diseased muscles, where different types of motor neurons are affected differently.

### SHG measurements are consistent with a biphasic loss of motor neurons

Prior studies reported two distinct phases of neuronal death in the SOD1G93A model of ALS. In the first phase, which occurs much before symptomatic onset, the FF motor neurons are affected^9,10,19^. We observed neither weight loss nor tremors in 12-week-old B6.SOD1G93A mice, but we saw atrophy of type IIb fibers, suggesting denervation similarly observed in past studies^20^. The denervation of FF motor units, which have the fastest contractile times^21^, likely caused the early changes we observed in the distributions of rise and half-relaxation times. These distributions shifted towards slower time values, and the composite twitch times at this presymptomatic time point showed significant differences. While we believe this early difference is due to motor neuron death, it is possible that B6.SOD1G93A mice have naturally slower motor units. Future studies are needed at earlier time points to rule out this possibility, and determine the earliest at which our method is capable of measuring a difference.

In later stages of disease, S and FR motor neurons have a capacity to sprout and reinnervate fibers that were previously innervated by a FF motor neuron; this leads to a higher proportion of fibers being innervated by FR and S motor neurons in SOD1G93A mice^9–11^. Although past studies reported a stable motor neuron innervation period, followed by a rapid denervation of FR motor units that coincided with the start of clinical symptoms^10^, we observed a stable region of contractile timing beyond the emergence of symptoms. There was a significant rise in contractile times only with the onset of more severe symptoms of weakness, at 21 weeks.

Notably, the ALS model we used differs from the traditional transgenic SOD1G93A mouse model as it has fewer copies of the SOD1G93A gene and shows a delayed disease onset and end stage^22^. Hence, our results suggest motor neuron loss in this model may not be as sudden and complete at the first appearance of clinical symptoms compared to the traditional SOD1G93A transgenic model. Studies of faster progressing ALS models are necessary to further test the capability of our imaging approach to detect changes in motor unit composition prior to symptomatic onset.

The second phase of motor neuron loss leaves only S motor units intact as FR motor neurons die off^9,10^. This phase is indicated by our histological data taken at 21 weeks (Fig. 2e,f), and manifests physiologically as a shift of the rise and half-relaxation times towards slower values (Fig. 1a,b). This increase in rise and half-relaxation times after a period of relative stability likely corresponds to the second episode of motor neuron death in studies that report two episodic periods of motor neuron death^10,19^.

### SHG microendoscopy is applicable to preclinical settings

Our results suggest SHG microendoscopy to be a good tool for investigating the health of motor units in animal models of disease. In mice, assays that measure open field locomotion, hindlimb muscle girth^22^ or gait^23^ provide valuable information, but they do not directly reflect disease processes at the motor unit level. Such microscopic information could provide a more direct means for guiding drug development.

Non-terminal applications of motor unit number estimation (MUNE) can be performed by electromyography^24^, but MUNE does not reveal which motor unit types remain. Knowledge of motor unit type composition is important because two muscles could have the same number of motor units, but if the ratio of fast to slow motor units is different the two muscles may be at different disease stages. Terminal experiments in mice have shown pre-symptomatic changes to contractile force and motor unit number^19,25^ that may reflect motor unit type. SHG microendoscopy provides a non-terminal means of assessing motor unit health that reflects the type of motor units remaining. Another new technology, Stimulated Raman Scattering (SRS) microscopy can detect early peripheral nerve degeneration *in vivo* in SOD1 mice before even EMG based methods^26^. However this requires a 1 cm incision to reveal the sciatic nerve. SHG microendoscopy is thus a less invasive procedure. Even in the relatively small muscles of the mouse, insertions of the 20-gauge probe had no discernible effect on motor unit contraction times. Hence, repeated tests of the same mouse is feasible, which enables longitudinal studies of drug efficacy with a powerful statistical design in which individual subjects are compared to themselves over time. Therapies that target patients with mutations of the SOD1 gene have successfully completed early clinical study phases, and have prolonged survival in SOD1 related animal models^27,28^. Microendoscopy measurements in animal models given these drugs should be performed to understand the effects of the drug on motor unit distribution over time in individuals, and further validate the measurement method.

Our method opens up the door for future studies of ALS models. Just by taking a handful of measurements from different mice in the gastrocnemious, we were able to determine a sensitive metric for diagnosing ALS presymptomatically. The gastrocnemius muscle was easily accessible by the microendoscope in a mouse model, but larger animal models may allow greater exploration of other muscles, as well as specific positioning of the probe relative to nerves, which may prove to be even more sensitive. Our efforts developing the composite twitch time shows that similar efforts in other muscles can lead to an easily interpretable metric for tracking ALS.

### SHG microendoscopy may be applicable to clinical settings

SHG microendoscopy appears promising for monitoring and diagnosing ALS through minimally invasive measurements of motor unit contractile dynamics in humans. While this study did not recruit human subjects, the technology has been used in humans to assess muscle dynamics after stroke^8^. Our measurement technique would compliment other proposed electrophysiological monitoring biomarkers of muscle in humans^29,30^. MUNE and MUNIX (Motor unit number index) is one class of techniques that estimates the number of motor units in a muscle through electromyography. Several MUNE techniques exist with different ways of estimating the size of a single motor unit, and each have sampling biases that lead to different estimates of numbers^31^. Most MUNE methods are applicable only to small muscles of the hands and feet, although some have been developed for use in proximal muscles such as the biceps^32^. Electrical Impedance Myography (EIM) is also an intriguing measure of muscle health, measuring significant changes in muscle impedance with progression of the disease in patients^33^. This technique is limited to superficial muscles where the electrode array can be applied over the skin. Our method could allow access to more muscles of interest and provides more direct data that was previously unobtainable. Tests of reproducibility in multicenter trails are crucial to the adoption of any physiological biomarker into clinical settings, along with evaluations of ease of use by clinicians and patient tolerance. Our results show that microendoscopy can provide contractile timing information, and many muscles of interest in the upper and lower limbs that may be inaccessible for MUNE or EIM may be targeted with our method. A combination of these approaches may prove to be the most beneficial clinically.

The ability to detect differences in populations at pre-symptomatic stages in mice suggests that it might be possible to improve early diagnosis in humans, allowing patients to participate in clinical trials and receive treatments earlier in the disease. Crucially, observations of motor unit twitch contractions using SHG microendoscopy is feasible in humans, and differences in contractile timing in different muscle types have been reported in healthy individuals^8^. Today, clinical and electrophysiological criteria have a ~76% diagnosis sensitivity for detecting ALS^34^. The inclusion of a metric for motor unit contractile times, such as our composite twitch time, may improve diagnostic sensitivity. As ALS affected the shape of the distributions of both the rise and half-relaxation times of motor units, a metric that captured both effects was a more sensitive biomarker for early diagnosis. Because the rise and half-relaxation times were faster at earlier weeks, the harmonic mean and median were more sensitive at early disease stages when faster motor units were denervated. Future studies should examine if the composite twitch time is equally sensitive in muscles that have different motor-unit type compositions from the mouse gastrocnemius, especially as human muscles typically have a more heterogeneous mix of motor unit types^35^. To evaluate accuracy of SHG microendoscopy based diagnosis, measurements in ALS patients are needed to confirm if human motor units become slower at disease onset as compared to the motor units of healthy humans.

Our results showcase SHG microendoscopy in measuring differences between wild-type and a diseased model of mice before overt symptoms and tracking the progression of the disease. By measuring motor unit twitch contractile dynamics in a minimally invasive manner and showing quantitative changes due to specific motor neuron loss that precedes clinical symptoms, we have provided an important new research and clinical tool for ALS and other neurodegenerative diseases.

## Methods

### Mice

All procedures were approved by the Stanford Administrative Panel on Laboratory Animal Care, and were performed in accordance with APLAC and NIH guidelines for care and use of laboratory animals. We used ten female B6.SOD1G93A mice (B6.Cg-Tg(SOD1*G93A)1Gur/J**;** Jackson Laboratory) and five female wild-type mice (C57BL/6; Taconic). We housed mice in groups of up to 5 in a 12 h /12 h light/dark cycle. We imaged mice at approximately 86, 105, 120, 135, 150, and 162 days of age. We imaged five B6.SOD1G93A mice across all six time points, except for one mouse, which reached disease end stage before the last time point and was euthanized. We also imaged a sixth B6.SOD1G93A mouse across the first five time points and excised its triceps surae muscles for histological analyses and euthanized it after the imaging. We imaged the five wild-type mice at all 6 time points (Fig. 1e).

### Mouse body masses

We weighed all mice while they were awake. The values plotted in Fig. 3e are mean values for all mice that were alive at each time point.

### Neurological scores

We scored B6.SOD1G93A mice as follows: 0: no symptoms; 1: trembling when suspended by tail; 2: weakness—a lack of ankle extension when suspended or foot-dragging during locomotion; 3: paralysis—minimal joint movement, foot not being used to generate power; 4: end stage—mouse cannot right itself within 5 s. We immediately euthanized any mouse that scored a 4. We scored the mice at least twice after scores of 0 or 1; at least 3 times a week after a score of 2; and at least every other day after a score of 3. In computations of average scores, once a mouse reached stage 4 we recorded its score as 4 for all successive time points. If a mouse was sacrificed without reaching a score of 4, we did not include the mouse in the computation of mean scores for subsequent time points.

### SHG imaging procedures

Prior to SHG imaging we anesthetized the mouse  with 2% isoflurane in O_2_ and placed it on a heating pad maintained at 30 °C. We removed fur from the posterior sides of the lower hindlimbs. We secured the mouse with its hindlimbs as extended as possible and with the hips square. The microendoscope probe consists of a 20-gauge hypodermic needle with a solid sharp tip, with a side viewing optical window near the tip. We then inserted the microendoscope probe into the belly of the gastrocnemius, approaching the muscle from the lateral side (Fig. 1b). To induce intramuscular stimulation, we connected the electrical stimulator to the microendoscope probe via its metal sheath and inserted a 30-gauge needle above the mouse’s ankle under the Achilles tendon to serve as the ground electrode. We began continuous SHG imaging while stimulating the muscle with negative-going voltage pulses (1 Hz frequency; 0.1 ms pulse duration) using a waveform generator. We started with pulses of –100 mV and increased the pulse amplitude in increments of −10 mV until a twitch response appeared in the SHG image. Maintaining the stimulation voltage at this threshold level, we recorded SHG images for at least 10 s. We then further increased the stimulation voltage, and recorded SHG images with each subsequent quantal jump in twitch amplitude, each corresponding to the addition of a motor unit^8^, up to a voltage of –10 V. If we were unable to induce any twitch responses with pulses 0.1 ms in duration, we increased the pulse duration up to 1 ms in increments of 0.1 ms. After evoking twitches, we turned off the stimulator and adjusted the position of the microendoscope probe by a few millimeters laterally. We repeated the process at least 3 times in each leg of each mouse. After imaging both legs, we allowed the mouse to recover back in the home cage. In some cases, we were unable to image twitch responses in some locations in the muscle, hence there are different numbers of measurement sites in each mouse. One B6.SOD1G93A mouse was sacrificed immediately after each time point and had at least 3 insertions into each leg. After each imaging session, we disinfected the microendoscope probes with ASP Cidex Ortho-Phthalaldehayde Solution.

### Analysis of motor unit contractile speeds

We extracted kinetic time constants from the line-scanning images of twitch contractions over time by fitting data points of displacement and time to a parameterized model of motor unit twitch contraction.

To obtain the displacement versus time data points for individual motor units, we first computed the averaged twitch image across the entire set of twitches imaged under the same voltage settings and position by temporally aligning the line scan recordings to the instant 50 ms before the start of each electrical pulse and lasting for 500 ms. Figure 1c,d show examples of averaged twitch images. We then further cropped and averaged the left side of the image, before stimulation, to one column that represents the resting muscle. We determined for each subsequent time point (column in the averaged twitch image) the vertical displacement resulted in the lowest sum of squared errors when compared to the resting column. If this automatic method did not provide a good visual match to the twitch image, we manually traced the displacement over times in the image. We excluded from analysis any twitch image that lacked a clearly visible maximum of displacement.

For the twitch at the lowest stimulating voltage, we fit these displacement versus time points, substituting displacement for force, directly to a parameterized model of motor unit twitch contraction with parameters reflecting the contraction time, half-relaxation time, and motor unit twitch force^21^, from which we calculate the rise and the half-relaxation times. The model is *F*(*t*) = *pt*^*m*^*e*^*−kt*^, where *p* = ${F}_{{\max }}{{\rm{e}}}^{-k{T}_{c}(\mathrm{ln}{T}_{c}-1)}$, *m* = *kT*_*c*_, and *k* = ln 2/(−*T*_*c*_ ln(*T*_*hr*_/*T*_*c*_) + *T*_*hr*_ –*T*_*c*_), where *T*_*c*_ is the time from force development to where force reaches maximum, *T*_*hr*_ is the time from force development to when the force decreases to *F*_*max*_/2, and *F*_*max*_ is the maximum force^21^. We allowed there to be a delay time from electrical stimulus to the time of force displacement in fitting the curve.

We measured additional motor units by subtracting the subsequent twitches recorded at higher voltages. To do so, from the averaged twitch image we traced the displacement of the twitch over time manually, by selecting the displacement at different time points across the image. At those time points, we subtracted the displacement value at that time point from the previous model of twitch. We then fit the resulting difference to the twitch model.

### Statistical analysis

We performed all statistical analyses in MATLAB (Mathworks) and used significance criterion of p < 0.05 for all tests. We used a Gore test for multiple observations to test for differences between the time points in the measured rise and half-relaxation times. We then performed a post-hoc Dunn’s test with the Bonferroni correction for multiple comparisons to determine which time points were different from others. We used a Kolmogorov–Smirnov test to check for differences in the distributions of rise and half-relaxation times between the ALS model and wild-type mice. We used a Gore test for multiple observations to test for differences in individual mice in the measured rise and half-relaxation times.

To visualize and estimate probability density functions, we used a kernel density estimator algorithm^36^ based on linear diffusion with automatic bandwidth selection. This method avoids the arbitrary selection of bin size required for histograms, and does not assume a parametric model for the data. We used kernel density estimation for improved visual display and not for statistical interpretation.

To analyze metrics related to the composite twitch time, we used a Wilcoxon rank sum test to assess the differences of the harmonic mean of the rise times and the median of the half relaxation between B6.SOD1G93A and wild-type mice at each experimental time point. We created receiver operating curves (ROCs) to analyze how well the composite twitch time served as a diagnostic metric of ALS. We determined confidence intervals on the AUC through 1000 bootstrap samples.

While the ROC analysis gave us a sense of how well the composite twitch time performs over all time points, to test for differences across the six time points, we used a Skillings–Mack test. We performed post-hoc Dunn’s test with a Bonferroni correction for multiple comparisons to determine which time points had different values than the others.

To determine if the harmonic mean of the rise time and the median half-relaxation time had the same dependence on the age of the B6.SOD1G93A mice, we first fit each dataset to a three-parameter function of the form *y* = *A*e^*Bx*^ + *C*, where *x* is the age of the mouse since the first time point, *C* is the mean of the data at the first time point, and *A* and *B* were parameters determined from a least-squares fit. We then performed a pair of F-tests to compare the reduced chi-squared values attained by applying the parameter values from the fit to one of the datasets and applying it to the other dataset.

### Immunohistochemical analyses

Following SHG microendoscopy we removed the triceps surae of the mouse and immediately placed the muscles in optical cutting temperature compound and froze them at −20 °C. We then cut 10 µm sections with a cryostat and mounted them on Superfrost slides.

We rinsed the slides in PBS and then applied blocking solution (0.3% Triton and 2% normal goat serum (NGS) dissolved in PBS) for 1 h. We double stained for either myosin heavy chain Type I and Type IIb, or Type IIa and Type IIx, by incubating the slides overnight with primary antibodies and concentrations of BA-D5 (1:100)^37^, BF-F3 (1:50)^37^, and SC-71 (1:500)^37^, and 6H1 (1:100)^38^, respectively, in 0.3% Triton and 5% NGS dissolved in PBS (all primary antibodies from Developmental Studies Hybridoma Bank). The next day, we rinsed the slides in PBS twice and incubated the slides for 2 h with 1:500 dilutions of secondary antibodies. We used Alexa Fluor 488 Goat Anti-Mouse IgG_1_ for SC-71, Alexa Fluor 488 Goat Anti-Mouse IgG_2b_ for BA-D5, and Alex Fluor 594 Goat Anti-Mouse IgM for 6H1 and BF-F3 (LifeTechnologies). We then rinsed the slides in PBS and attached cover slips with PVA-Dabco. We stored the slides at −20 °C.

We imaged the tissue slides using a Leica DM4000 B fluorescence microscope equipped with 5× (Leica N PLAN 5 × /0.12 PH0) and 10× (Leica HCX PL Fluotar 10 × /0.30 PH1) objective lenses and a MBF CX9000 camera. We computationally stitched together the images taken with the 5× objective using the image analysis software Fiji, a distribution of ImageJ^39^.

## Supplementary information

Supplementary Material

## Data Availability

The datasets generated and analyzed during the current study are available from the corresponding authors on reasonable request.
