# Supplementary material for: Microendoscopy detects altered muscular contractile dynamics in a mouse model of amyotrophic lateral sclerosis
Source: Sci Rep. 2020 Jan 16;10:457. doi: 10.1038/s41598-019-56555-z (PMC6965652; doi:10.1038/s41598-019-56555-z)
Supplement: Supplementary file 1 — Supplementary Material [file 41598_2019_56555_MOESM1_ESM.docx]

**Microendoscopy detects altered muscular contractile dynamics in a mouse model of amyotrophic lateral sclerosis**

Xuefeng Chen^1^, Gabriel N. Sanchez^5,6^, Mark J. Schnitzer^2,3,4,*^, and Scott L. Delp^1,5,*^

*^1^Dept. of Mechanical Engineering, ^2^Dept. of Biology, ^3^Dept. of Applied Physics, ^4^Howard Hughes Medical Institute, ^5^Dept. of Bioengineering, Stanford University, Stanford CA USA 94305.*

*^6^Enspectra Health, Mountain View CA USA 94040.*

*Correspondence: delp@stanford.edu, schnitzer@stanford.edu

**Supplementary Figure 1. Microendoscope insertion does not affect muscle force generation.**


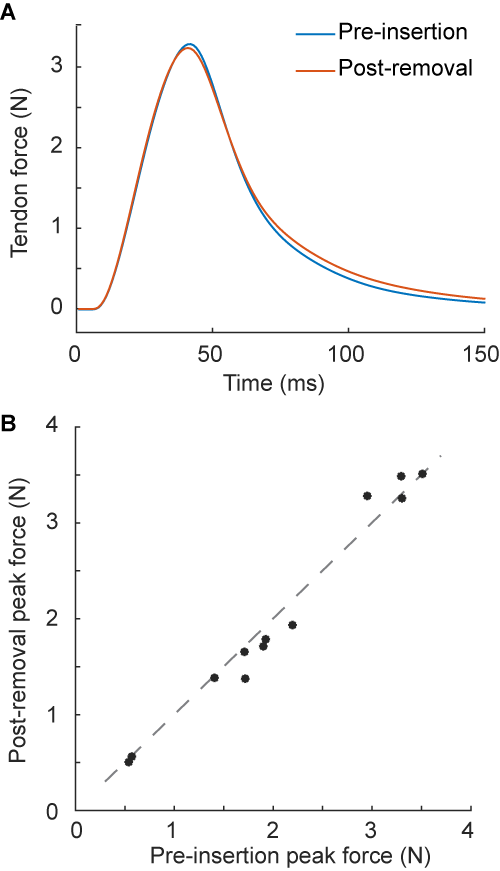


While mice were under anesthesia, we isolated the Achilles tendon, cut the calcaneus and attached a force transducer to the calcaneus bone piece. We exposed the sciatic nerve, placed an electrical cuff attached to a signal generator around the nerve, and elicited a series of ten twitches evoked at 1 Hz. The 20-gauge microendoscope probe was inserted for 10 s, then removed, and another 10 twitches were recorded with the same stimulation parameters. After 2 min, the process was repeated, up to 3 times.

**(A)** Example traces of average twitch tendon force, acquired from the same muscle just before insertion of the microendoscope probe (*red*), and immediately after microendoscope removal (*blue*).

**(B)** Peak twitch forces before insertion of the microendoscope and after its removal (p = 0.18, Wilcoxon signed rank test, n = 4 mice, 12 trials total). Error bars (s.e.m.) are not visible because they are smaller than the data markers. Dashed diagonal line is the line of equality.

**Supplementary Figure 2. Measured twitch contractile values from individual motor units**


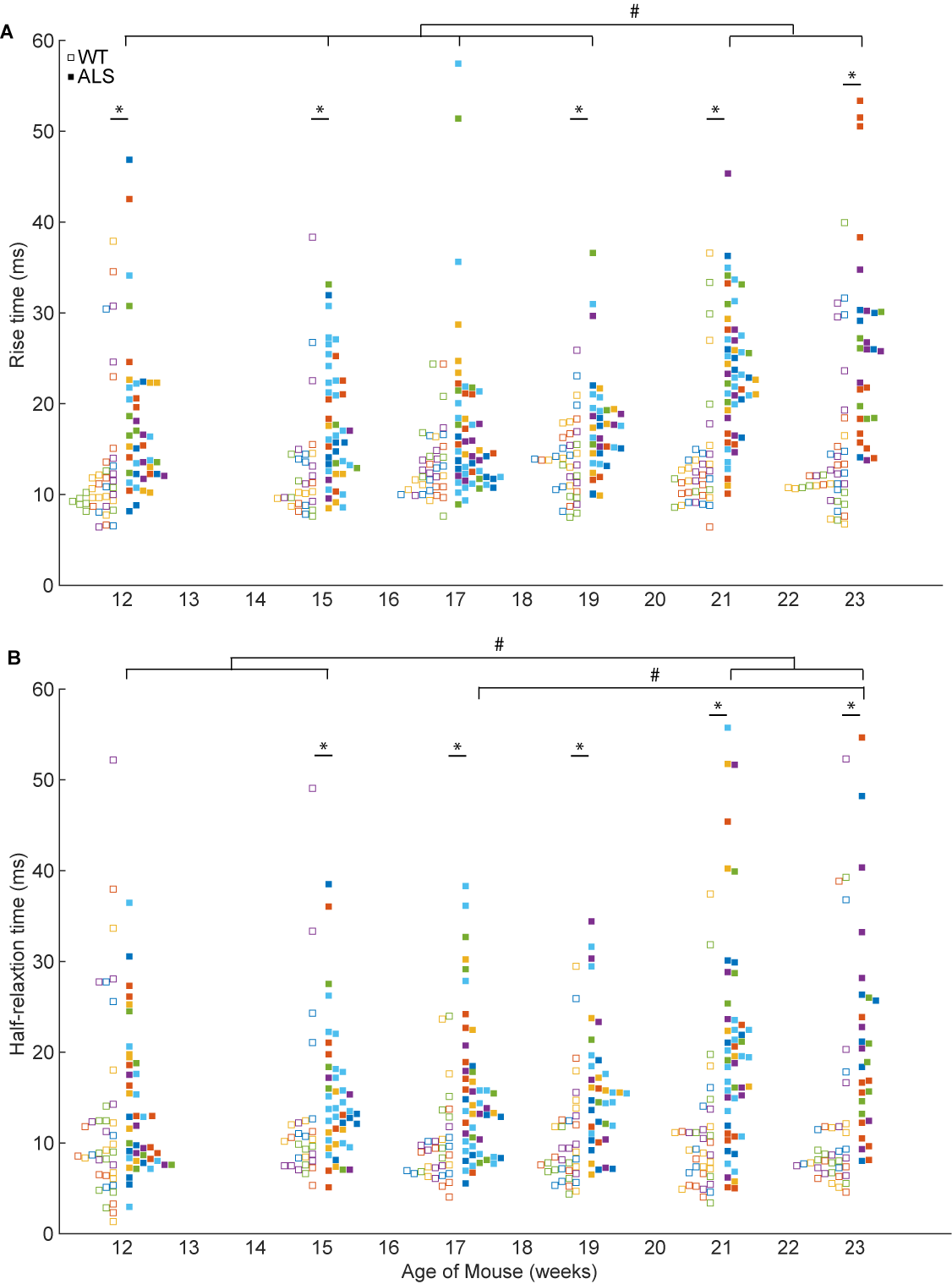


**(A)** All rise times recorded from individual motor units in all mice.

**(B)** All half-relaxation times recorded from individual motor units in all mice.

Different colors at each time point are from different mice. Same color data points are different motor units within a mouse, measured from different positions or stimulation voltages. * p < 0.05, Kolmogorov–Smirnov test. # p < 0.05, Dunn’s test for multiple comparisons with Bonferroni correction. n = 29–54 motor units at each time point from each mouse type.

**Supplementary Table. Evaluations of features for diagnosis and monitoring. Related to Fig. 3.**

|  | Sum p-values | ROC AUC  all time points | ROC AUC  first 2 time points | *r* |
| --- | --- | --- | --- | --- |
| Harmonic mean *t_r_* | **0.18** | 0.92 | 0.86 | **0.69** |
| Median *t_r_* | 0.19 | 0.90 | 0.83 | 0.63 |
| 25% *t_r_* | 0.22 | 0.90 | 0.80 | 0.72 |
| 25% *t_hr_* | 0.22 | 0.93 | 0.87 | 0.64 |
| Harmonic mean *t_hr_* | 0.24 | 0.93 | 0.87 | 0.67 |
| Mean *t_r_* | 0.26 | 0.87 | 0.83 | 0.63 |
| Minimum *t_r_* | 0.34 | 0.93 | 0.86 | 0.56 |
| Median *t_hr_* | 0.40 | **0.94** | **0.88** | 0.67 |
| 75% *t_r_* | 0.69 | 0.82 | 0.76 | 0.57 |
| Mean *t_hr_* | 0.77 | 0.88 | 0.79 | 0.70 |
| Minimum *t_hr_* | 0.86 | 0.84 | 0.78 | 0.35 |
| Maximum *t_hr_* | 1.49 | 0.70 | 0.60 | 0.47 |
| 75% *t_hr_* | 1.63 | 0.84 | 0.75 | 0.61 |
| Standard deviation *t_hr_* | 2.09 | 0.64 | 0.57 | 0.44 |
| Mean ratio *t_r_* / *t_hr_* | 2.23 | 0.29 | 0.38 | -0.20 |
| Maximum *t_r_* | 2.37 | 0.69 | 0.66 | 0.18 |
| Skewness *t_hr_* | 2.84 | 0.45 | 0.46 | 0.03 |
| Skewness *t_r_* | 3.22 | 0.43 | 0.55 | -0.39 |
| Standard deviation *t_r_* | 3.73 | 0.61 | 0.64 | 0.04 |

To determine the best metric for identifying the changing distribution of motor unit twitch time constants in ALS mice, we analyzed features constructed from the rise and half-relaxation times measured in each mouse. The features were the mean, standard deviation, harmonic mean, skewness, all quartiles, and minimum and maximum values for the rise and the half-relaxation times, as well as the mean of the ratio of the rise to half-relaxation times. We evaluated these 19 features based on the sum of the p-values when performing a rank-sum test comparing B6.SOD1G93A and WT mice at all 6 time points, the AUC of the ROC analysis when including data from all time points, and the AUC of a ROC based on B6.SOD1G93A data from the first two time points and all of the wild type time points. The features listed in Table S1 are with the highest performing metric shown in bold. As the median and the harmonic mean of the rise and half relaxation times performed well, we performed a leave-one-out cross validation on a logistic-regression model based on these features. The AUC under the ROC for the leave-one-out data for the harmonic mean of rise time plus the median of the half-relaxation time was 0.95. The AUC for median rise time plus median half relaxation time was 0.94. The AUC for harmonic mean of rise time plus harmonic mean of half relaxation time was 0.94. Based the largest resulting AUC, we chose the composite twitch time to be the harmonic mean of the rise time plus the median half-relaxation time.
